# Gene-wide Association Study Reveals *RNF122* Ubiquitin Ligase as a Novel Susceptibility Gene for Attention Deficit Hyperactivity Disorder

**DOI:** 10.1038/s41598-017-05514-7

**Published:** 2017-07-14

**Authors:** Iris Garcia-Martínez, Cristina Sánchez-Mora, María Soler Artigas, Paula Rovira, Mireia Pagerols, Montse Corrales, Eva Calvo-Sánchez, Vanesa Richarte, Mariona Bustamante, Jordi Sunyer, Bru Cormand, Miquel Casas, Josep Antoni Ramos-Quiroga, Marta Ribasés

**Affiliations:** 1 Psychiatric Genetics Unit, Group of Psychiatry, Mental Health and Addiction, Vall d’Hebron Research Institute (VHIR), Universitat Autònoma de Barcelona, Barcelona, Catalonia Spain; 2 Department of Psychiatry, Hospital Universitari Vall d’Hebron, Barcelona, Catalonia Spain; 3 Biomedical Network Research Centre on Mental Health (CIBERSAM), Instituto de Salud Carlos III, Madrid, Spain; 4 Department of Psychiatry and Legal Medicine, Universitat Autònoma de Barcelona, Barcelona, Catalonia Spain; 5 ISGlobal, Centre for Research in Environmental Epidemiology (CREAL), Barcelona, Spain; 6 Genomics and Disease Group, Bioinformatics and Genomics Program, Centre for Genomic Regulation (CRG), Barcelona, Spain; 7 Universitat Pompeu Fabra (UPF), Barcelona, Spain; 8 CIBER Epidemiología y Salud Pública (CIBERESP), Madrid, Spain; 9 IMIM (Hospital del Mar Medical Research Institute), Barcelona, Spain; 10 Departament de Genètica, Microbiologia i Estadística, Facultat de Biologia, Universitat de Barcelona, Barcelona, Catalonia Spain; 11 Centro de Investigación Biomédica en Red de Enfermedades Raras (CIBERER), Instituto de Salud Carlos III, Madrid, Spain; 12 Institut de Biomedicina de la Universitat de Barcelona (IBUB), Barcelona, Catalonia Spain; 13 Institut de Recerca Sant Joan de Déu (IR-SJD), Esplugues, Catalonia Spain

## Abstract

Attention Deficit Hyperactivity Disorder (ADHD) is a common childhood-onset neurodevelopmental condition characterized by pervasive impairment of attention, hyperactivity, and/or impulsivity that can persist into adulthood. The aetiology of ADHD is complex and multifactorial and, despite the wealth of evidence for its high heritability, genetic studies have provided modest evidence for the involvement of specific genes and have failed to identify consistent and replicable results. Due to the lack of robust findings, we performed gene-wide and pathway enrichment analyses using pre-existing GWAS data from 607 persistent ADHD subjects and 584 controls, produced by our group. Subsequently, expression profiles of genes surpassing a follow-up threshold of P-value < 1e-03 in the gene-wide analyses were tested in peripheral blood mononucleated cells (PBMCs) of 45 medication-naive adults with ADHD and 39 healthy unrelated controls. We found preliminary evidence for genetic association between *RNF122* and ADHD and for its overexpression in adults with ADHD. *RNF122* encodes for an E3 ubiquitin ligase involved in the proteasome-mediated processing, trafficking, and degradation of proteins that acts as an essential mediator of the substrate specificity of ubiquitin ligation. Thus, our findings support previous data that place the ubiquitin-proteasome system as a promising candidate for its involvement in the aetiology of ADHD.

## Introduction

Attention Deficit Hyperactivity Disorder (ADHD) is a common childhood-onset neurodevelopmental disorder with a high estimated prevalence of 5.3% among children and of 2.5% in adulthood^1^. Family and twin studies have shown that genetic factors play a crucial role in ADHD susceptibility and have estimated the heritability of the disorder to be around 76–80% both in children and in adults^1^.

In spite of this high heritability, genome-wide linkage studies or hypothesis-driven candidate gene association analyses in ADHD have failed to identify consistent and replicable genetic factors, and provide modest evidence for the involvement of some specific genes on the basis of meta-analyses^2–5^. Aiming to overcome these issues, and along with advances in high-throughput technologies, a number of genome-wide association studies (GWAS) has been performed in ADHD in the last few years. It has been reported that around 28% of the total variance in the liability to ADHD may be explained by common nucleotide polymorphisms (SNPs), and that a considerable part of this estimated SNP-based heritability might be shared with different psychiatric disorders^6, 7^. GWAS in ADHD have shown suggestive evidence for association of cadherin 13 (*CDH13*) and other candidate genes, including monoamine system-related genes (e.g. *SLC9A9* and *SNAP25*), the glutamate metabotropic receptor 5 (*GRM5*), the glucose-fructose oxidoreductase domain containing 1 (*GFOD1*), the cannabinoid receptor 1 (*CNR1*), the protein kinase CGMP-dependent 1 (*PRKG1*) or the nicotinic acetylcholine receptor alpha 7 (*CHRNA7*), among others^2–4, 8^. However, no genome-wide significant findings have been identified [P≤5e-8], there is limited overlap between results from different GWAS, and none of the classical candidate genes for ADHD has been found among the top findings. These results may reflect differences in study design or phenotypes analyzed in the different GWAS performed so far, and suggest that larger samples, more homogeneous populations and more complex statistical strategies are required to identify genetic variants displaying low to moderate effects in ADHD^4, 8^.

Considering the absence of genome-wide significant associations and the limited overlap between top hits from SNP-based GWAS on ADHD, analyses focusing on gene-set enrichment or pathway-based approaches raise as promising strategies to address the genetic complexity of the disorder^9^. These strategies allow combining effects of multiple SNPs (gene-wide studies) or multiple genes (gene-set analyses), while reducing multiple testing comparisons and providing insights into the involvement of specific biological functions or pathways^9^. In this line, Mick *et al*. performed gene-wide analyses and provided additional evidence for the association between ADHD and the solute carrier *SLC9A9*, a candidate gene for the disorder that had been found nominally associated with ADHD in other SNP-based GWAS^2–4, 8^. Gene-set enrichment approaches or pathway analyses on ADHD also highlighted genes involved in the regulation of gene expression, cell adhesion and inflammation, ubiquitin-mediated proteasomal degradation, neurodegenerative disorders, axon guidance, neuron projections or synaptic components, and revealed significant overlap between pathways enriched for SNP association and those enriched for rare copy number variants (CNV)^2–4, 8^. To integrate findings from GWAS and provide knowledge about potential molecular processes underlying ADHD, Poelmans *et al*. performed network analysis considering top findings from five GWAS and revealed that 45 of the 85 top-ranked ADHD candidate genes encoded proteins that fitted into a neurodevelopmental network involved in directed neurite outgrowth^10^. Recently, the Psychiatric Genomics Consortium (PGC) combined GWAS signals from over 60,000 subjects to identify common network and biological pathways across three adult psychiatric conditions: schizophrenia, major depression and bipolar disorder. This study highlighted an important overlap across disorders, with histone methylation processes showing the strongest association, as well as multiple immune and neuronal signaling pathways^11^.

Due to the lack of robust findings in ADHD through SNP-based GWAS, which may be explained by the lack of power of the studies conducted so far and the polygenic, multifactorial nature of the disorder -with common and rare variants likely contributing small to moderate effects to its etiology-, we performed gene-wide and pathway enrichment analyses using GWAS data from 607 persistent ADHD subjects and 584 controls. Subsequently, expression profiles of genes surpassing a follow-up significance threshold of P-value < 1e-03 in the gene-wide analyses were tested in peripheral blood mononucleated cells (PBMCs) of 45 medication-naive adults with ADHD and 39 healthy unrelated controls.

## Materials and Methods

### Subjects and Clinical Assessment

The clinical sample consisted of 607 adult ADHD subjects and 584 unrelated healthy individuals, all Spanish and Caucasian. Detailed information regarding the sample is described elsewhere^12^.

The evaluation of the ADHD diagnosis was carried out with the Structured Clinical Interview for DSM-IV Axis I and II Disorders (SCID-I and SCID-II) and with the Conners’ Adult ADHD Diagnostic Interview for DSM-IV (CAADID parts I and II)^13^. Severity of ADHD symptoms in adulthood was assessed with the long version of the Conners’ ADHD Rating Scale (self-report [CAARS-S:L] and observer [CAARS-O:L])^14^, the ADHD Rating Scale (ADHD-RS)^15^ and the Wender Utah Rating Scale (WURS) for retrospective symptomatology in childhood^16^. The level of impairment was measured with the Clinical Global Impression (CGI) and the Sheehan Disability Inventory^17, 18^. Additional tests used for clinical assessment are available in Ribasés *et al*.^19^. Exclusion criteria were IQ < 70; lifelong and current history of mood, psychotic, anxiety, substance abuse, and DSM-IV axis II disorders; pervasive developmental disorders; a history or the current presence of a condition or illness, including neurologic, metabolic, cardiac, liver, kidney, or respiratory disease; a chronic medication of any kind; birth weight ≤ 1.5 kg; and other neurological or systemic disorders that might explain ADHD symptoms.

The control sample consisted of unrelated healthy individuals matched for sex with the clinical group. ADHD symptomatology was excluded retrospectively under the following criteria: 1) not having been diagnosed with ADHD previously and 2) answering negatively to the life-time presence of the following ADHD symptoms: a) often has trouble in keeping attention on tasks; b) usually loses things needed for tasks; c) often fidgets with hands or feet or squirms in seat and d) often gets up from seat when remaining in seat is expected.

All subjects were evaluated and recruited at Hospital Universitari Vall d’Hebron of Barcelona (Spain) and diagnosis was blind to genotype. The study was approved by the Clinical Research Ethics Committee (CREC) of Hospital Universitari Vall d’Hebron, all methods were performed in accordance with the relevant guidelines and regulations and written informed consent was obtained from all subjects before the inclusion into the study.

### Gene-wide Analysis

Genome-wide genotyping of 607 adults with ADHD and 584 healthy controls was performed with the Illumina HumanOmni1-Quad BeadChip platform [Illumina Inc., San Diego, California, USA]. Quality control assessment was implemented at the individual and SNP level using PLINK v1.07^20^ and included filtering subjects with low call rate (<98%) or gender discrepancy, followed by filtering SNPs with minor allele frequency (MAF) < 0.01, Hardy-Weinberg equilibrium test P-values (P_HWE_) < 1e-06 or call rate <0.99 in either cases or controls. After stringent quality control assessment, five samples were excluded from the analysis due to low call rate and a total of 794,090 SNPs with a mean call rate of 0.9994 for the remaining 603 cases and 583 controls were included in the study.

Genome-wide association analysis was performed using the Cochran-Armitage trend test with PLINK v1.07^20^ and the genomic inflation factor (λ) of 1.031 was used to correct for the degree of inflation^12^. Gene-wide association analyses were conducted with the VEGAS software [VErsatile Gene-Based Association Study; http://gump.qimr.edu.au/VEGAS/], which combines the effect of all SNPs in a gene locus and corrects for linkage disequilibrium (LD)^21^. Regions were defined using VEGAS default option as ±50 kb upstream and downstream from each gene locus (UCSC Genome Browser; NCBI36/hg18 assembly), and the HapMap CEU population was used to estimate patterns of LD for each gene. Two different strategies were followed for gene-wide analyses by (i) considering all SNPs that mapped within a gene locus and (ii) restricting the analysis to the 10% most significant SNPs from each gene locus. Multiple-testing adjustment was addressed by the Bonferroni correction, and the significance threshold was set at P-value < 1.4e-06, taking into account 17,787 autosomal genes and the two gene-wide strategies (0.05/(17,787*2)). Positive signals surpassing a follow-up threshold of P-value < 1e-03 were tested in an independent dataset of 2,064 ADHD trios, 896 ADHD cases and 2,455 controls, following the same strategy described for the discovery sample^7, 22^.

### Gene-Set and Pathway Analysis

Gene-set and pathway analyses were restricted to genes surpassing a follow-up significance threshold set at P-value < 1e-03 in the gene-wide analyses when either all SNPs or the 10% most significant SNPs in gene locus were considered. The definition of the follow-up threshold was based on previous evidence supporting that VEGAS software provides an accurate performance for genes with P-value < 1e-03, offering a sizeable sensitivity, with less than 1% false positives and specificities ranging from 98% to 100%, while being able to distinguish between multiple independent causal loci and multiple signals due to linkage disequilibrium^23^.

The gene-set analyses were performed with MAGMA software [Generalized Gene-Set Analysis of GWAS Data; http://ctg.cncr.nl/software/magma]^24^, a statistical method for analyzing multiple genetic variants simultaneously to determine their joint effect while correcting for LD (gene-wide), and subsequently, assembling individual genes into groups of genes sharing biological or functional characteristics (gene-set). Multiple-testing correction was assessed by 10,000 permutations as implemented in the MAGMA software and the significance threshold was set at adjusted P-value < 0.05.

Functional and pathway enrichment analyses were conducted with IPA software [Ingenuity Pathway Analysis; Ingenuity Systems®, Redwood City, California, USA; http://www.ingenuity.com]. Fisher’s exact test was used to calculate P-values, based on the number of genes/molecules that map to a biological function, disease, pathway, or network. A Benjamini-Hochberg’s (BH) threshold of 0.05 was applied for multiple comparison correction in the functional, disease and pathway enrichment analyses. Gene networks were considered of relevance when the network score (P-score = −log10(P-value)) was over 8 (P-value < 1e-08)^25, 26^.

### Gene expression analysis in peripheral blood mononuclear cells (PBMCs) with microarrays

Expression levels of genes identified in the gene-wide study (follow-up P-value threshold <1e-03) were tested in PBMCs of 45 medication-naive adults with a new clinical diagnosis of ADHD (69% male; mean age = 38 years (SD = 9.9)) and 39 healthy unrelated controls (60% males, mean age = 35 years (SD = 12.1)).

Briefly, PBMCs were isolated using the Ficoll density gradient method, and total RNA was extracted using Qiazol Lysis reagent and the RNeasy Midi kit [Qiagen, Hilden, Germany]. The quality of the samples was assayed by 2100 Bioanalyzer [Agilent Technologies Inc. Santa Clara, California, USA]. RNA was reverse transcribed using the Ambion WT Expression Kit [Life technologies, Massachusetts, USA]. The cRNA was subsequently fragmented, labelled and hybridized with the GeneChip WT Terminal Labeling and Hybridization Kit to the Genechip Human Gene 1.1 ST 96-Array plate [Affymetrix, Santa Clara, California, USA]. The experiment was performed in 9 different amplification rounds, introducing a batch factor that was taken into consideration in the following statistical analyses. The array processing and data generation were assessed using the Gene Titan Affymetrix microarray platform. Background correction, normalization and summarization of probes values was performed using the Robust Multichip Average (RMA) algorithm implemented in the *oligo R* library^27^. Expression patterns for genes of interest in ADHD subjects and controls were contrasted using the *limma* R library^28^, including batch and gender as covariates. Bonferroni correction was applied and the significance threshold was set at P-value = 1.5e-03, taking into account the 33 genes displaying P-values below the follow-up threshold in the gene-wide analyses and with microarray data available (0.05/33).

### Validation of gene expression differences with reverse transcription real-time quantitative polymerase chain reaction (RT-qPCR)

Validation of gene expression differences was performed on genes showing tentative evidence for differential expression in the microarray analysis, which included *MAB21L2*, *STXBP3* and *RNF122*. Gene expression validation assays were conducted with reverse transcription real-time quantitative polymerase chain reaction (RT-qPCR) in the same clinical sample of 45 ADHD cases and 39 controls. First, 2 µg of total RNA was reverse transcribed using the High Capacity cDNA Reverse Transcription kit according to manufacturer’s protocol in a final reaction volume of 20 µL [Applied Biosystems; Foster City, California, USA]. Following, RT-qPCRs were run in triplicate using 2 µL of cDNA, 2 µL of RNAse free water, 5 µL of TaqMan® Gene Expression Master Mix and 1 µL of TaqMan® Gene Expression Assay, in a final reaction volume of 10 µL [Applied Biosystems; Foster City, California, USA]. Reactions were measured in an Applied Biosystems 7900HT Fast Real-Time PCR system using the default thermal cycling conditions specified by the manufacturer [Applied Biosystems; Foster City, California, USA]. The threshold cycle (C_T_) was defined as the fractional cycle number at which the fluorescence exceeded the threshold of 0.2. The relative quantification of mRNA expression was calculated by the 2^−∆∆CT^ method^29^, considering only those samples showing standard deviations values ≤ 0.3 among triplicates and the *GADD45A* gene as an endogenous control, after checking its stability and linearity across all samples. Using the *Stats* R package [https://www.R-project.org/]^30^, generalized linear models (GLM) were applied to compare gene expression levels between ADHD cases and controls, including gender as covariate in the fitted model. The statistical test was one-sided and the Bonferroni correction was applied for multiple-testing control, setting the statistical significance threshold at P-value < 0.017 when taking into account three genes (0.05/3).

### Imputation, *cis*-expression Quantitative Trait Loci (*cis*-eQTL) Analyses and Prediction of Functional Effects

To better delineate the involvement of *RNF122* in ADHD and to detect potential functional variants, markers at this locus were imputed in the original dataset of 603 subjects with ADHD and 583 healthy controls. Pre-imputation quality control of the GWAS dataset at the individual and SNP level was implemented in accordance to the QC module instructions from the Ricopili pipeline considering default settings [https://sites.google.com/a/broadinstitute.org/ricopili/]. Screening for cryptic relatedness and population stratification was performed by Principal Components Analysis (PCA). Markers at the *RNF122* gene region plus 10 kb upstream and 5 kb downstream from the locus (chr8:33519815–33554185; NCBI36/hg18) were imputed in the GWAS sample through the pre-phasing and imputation strategies implemented by SHAPEIT and IMPUTE2, respectively^31, 32^, using the Ricopili pipeline [https://sites.google.com/a/broadinstitute.org/ricopili/] and data from the 1000 Genomes Project as the reference panel [http://www.1000genomes.org/]^33^. After filtering SNPs with MAF <0.01 and low imputation quality (r < 0.4), 138 SNPs were finally considered. We performed the association analysis using logistic regression models with the PLINK v1.07 software^20^ and multiple-testing was addressed by the Bonferroni correction, setting the significance threshold at P-value < 3.6e-04 when considering 138 imputed SNPs in the *RNF122* locus (0.05/138). Since this approach may be too conservative, alternative multiple-testing control was assessed using the Single Nucleotide Polymorphism Spectral Decomposition (SNPSpD) software [http://neurogenetics.qimrberghofer.edu.au/SNPSpDlite/]^34^, which takes into account patterns of LD (P-value<1.27e-03). Once top signals were identified, in order to uncover additional independent effects and to assess evidence for multi-risk loci in each region, further conditioned analysis was performed with PLINK v1.07 software^20^. To condition the logistic regression analysis on a specific SNP, we tested all markers again but adding the allelic dosage for the conditioned SNP as a covariate^20^.

The *cis-*eQTL analyses were conducted using genotype and expression data from a series of neuropathologically and neuropsychiatrically normal human brain samples from GSE8919^35^ and GSE30272^36^ datasets, available at the Gene Expression Omnibus site [http://www.ncbi.nlm.nih.gov/geo]. Genotype data from both studies were imputed following the methodology described above. Expression levels of the *RNF122* transcript NM_024787.2 were available for 62 cortical samples of European ancestry in the GSE8919 dataset (probe ID: GI_38045930-S) and for 94 prefrontal cortex samples of Caucasian origin in the GSE30272 dataset (probe ID:HEEBO-062-HCC62D14)^35, 36^. Rank-based inverse normal transformation of expression data was applied using *Stats* R package [https://www.R-project.org/]^30^ and additive linear regression models were fitted for eQTL mapping using PLINK v1.07 software^20^, considering covariates showing suggestive association with the outcome (P-value < 0.2; gender, age_at_death and transcripts_detected_rate24354 (average transcript detection rate for sample, out of all 24354 probes) for GSE8919 and sv2 (surrogate variable 2) for GSE30272 datasets). Bonferroni correction was applied for multiple testing and the significance threshold was set at P-value < 4.3e-04 for the GSE8919 dataset and at P-value < 3.8e-04 for the GSE30272 dataset, considering the number of available SNPs.

Potential functional effects of the independent risk alleles associated with ADHD were predicted using the SNPinfo, ESEfinder, and RESCUE_ESE web softwares^37–40^.

## Results

### Gene-Wide and Gene-Set Analysis

After individual and SNP-based standard quality control filtering, 794,090 autosomal SNPs in 603 adult ADHD cases and 583 healthy controls were included in the GWAS. The quantile–quantile plot showed no departure from the expected P-values distribution, with a genomic control inflation factor of λ = 1.031^12^.

The gene-wide association analysis revealed 20 genes surpassing the follow-up significance threshold (P-value < 1e-03) when considering all SNPs located within a gene locus, with *CTAGE5* (P-value <1.0e-06) and *FBXO33* (P-value = 3.0e-06) as the most associated genes (Table 1). Twenty additional genes were found associated with persistent ADHD when we considered the 10% most significant SNPs of each gene locus, with *KCNG4* (P-value = 1.0e-04) and *TAF1C* (P-value = 1.9e-04) as top signals (Table 1). After Bonferroni correction, only *CTAGE5* remained associated with ADHD. Consistently, the gene-set analysis revealed that both gene-sets were significantly associated with persistent ADHD after correcting for multiple comparisons (gene-set corrected P-value = 1e-03 when considering all SNPs within a gene locus and gene-set corrected P-value < 1e-03 when considering the top 10% SNPs in each locus) (Table 1), although no overlap was detected between them. However, some genes highlighted in the previous single-marker GWAS were found among top genes surpassing the follow-up significance threshold in the present gene-wide analyses (*FBXO33*, *PEX19*, *COPA* and *KCNG4*)^12^.

**Table 1:** Gene-wide and gene-set P-values considering all SNPs or the 10% most significant SNPs in gene locus using VEGAS and MAGMA softwares. Genes displaying P-value < 1e-03 are shown.

Chr	Gene	Number of SNPs	Start Position	End Position	Gene-wide P-value	Loci associated with neuropsychiatric or neurological disorders
**Full set of SNPs**						
**Global gene-set P-value = 2.2e**-**4 (P**_**corrected**_ ** = 1.0e**-**03)**						
14	*CTAGE5*	39	38754226	38940148	0*	Antidepressant efficacy in major depressive disorder, mild intellectual disability, and traits of inattention and hyperactivity^61, 66^
14	*FBXO33*	17	38886709	39021371	3.00e-06	Autism spectrum disorders, attention deficit, hyperactivity and mild mental retardation^12, 65, 66^
14	*MIA2*	26	38722875	38842326	4.00e-06	Mild intellectual disability, and traits of inattention and hyperactivity^61^
1	*PEX19*	10	158463225	158571555	3.40e-05	Major depressive disorder and ADHD^12, 67^
1	*COPA*	32	158475000	158629978	4.40e-05	Bipolar Disorder and ADHD^12, 68^
14	*PNN*	24	38664137	38772173	6.70e-05	Mild intellectual disability, and traits of inattention and hyperactivity^61^
1	*WDR42A*	34	158402128	158548603	8.80e-05	—
1	*NCSTN*	32	158529686	158645366	1.76e-04	Alzheimer’s Disease and Schizophrenia^69, 70^
1	*MAST2*	34	45991871	46324383	3.35e-04	—
1	*CASQ1*	46	158376988	158488300	3.56e-04	—
15	*CALML4*	14	66220096	66335502	4.50e-04	Major depressive disorder and stress^71, 72^
1	*ZP4*	45	236062332	236170558	5.44e-04	ADHD^73, 74^
1	*IPP*	22	45886993	46034720	6.75e-04	Cocaine dependence^75^
1	*TMEM69*	19	45876433	45982695	7.12e-04	Amphetamine effects and aggressiveness^76, 77^
4	*MAB21L2*	15	151672752	151775295	7.60e-04	Autism spectrum disorders, and mild intellectual disability^78–80^
1	*GPBP1L1*	29	45815567	45949398	7.75e-04	Autism spectrum disorders, and traits of inattention in Tourette syndrome^81, 82^
1	*STXBP3*	69	109040807	109203671	8.53e-04	—
13	*MLNR*	16	48642474	48744514	9.24e-04	—
2	*NLRC4*	26	32253021	32394305	9.36e-04	Schizophrenia, and bipolar disorder^83, 84^
1	*PEA15*	37	158391750	158501786	9.42e-04	Major depressive disorder^85^
**Top 10% of SNPs**						
**Global gene-set P-value = 1.12e**-**10 (P**_**corrected**_ ** < 1.0e**-**03)**						
16	*KCNG4*	118	82763323	82880857	1.00e-04	Alcohol consumption, and bipolar disorder in Amish population^86, 87^
16	*TAF1C*	86	82718961	82828163	1.90e-04	Autism and schizophrenia^88–90^
22	*NCF4*	48	35536975	35654005	2.10e-04	Schizophrenia^91^
22	*CSF2RB*	61	35589620	35716425	3.60e-04	Schizophrenia, major depressive disorder and bipolar disorder^92–94^
17	*AKAP10*	18	19699341	19871721	3.80e-04	Autism spectrum disorders, schizophrenia and bipolar disorder^95–97^
1	*ATP1A1*	23	116667358	116798919	4.10e-04	Major depressive disorder, autism spectrum disorders, anxiety and bipolar disorder^98–101^
8	*DUSP26*	21	33518392	33626981	5.10e-04	Atidepressant Efficacy in Major Depressive Disorder^66^
12	*RAP1B*	18	67240918	67390641	5.80e-04	Autism spectrum disorders^102, 103^
19	*AKAP8*	9	15275334	15401603	6.10e-04	Autism spectrum disorders^95, 104^
20	*RIN2*	113	19768209	19981100	6.40e–04	Bipolar dsorder, schizophrenia and stress-induced changes^105–107^
2	*SPAST*	29	32092183	32286210	6.80e-04	Autism spectrum disorders, and cognitive deficiency^109, 110^
7	*CCL26*	34	75186777	75307000	7.10e-04	Schizophrenia, and bipolar disorder^111, 112^
20	*NAT5*	59	19895936	20012269	7.60e-04	Autism spectrum disorders and comorbid anxiety^113^
8	*RNF122*	31	33474814	33594185	7.70e-04	Antidepressant efficacy in major depressive disorder^66^
15	*C15orf53*	66	36726090	36829531	7.90e-04	Bipolar disorder, and alcohol dependence^108, 114^
7	*GPR146*	34	1013666	1115423	8.90e-04	Autism spectrum disorders^115, 116^
8	*C8orf41*	25	33425777	33540245	9.20e-04	Antidepressant efficacy in major depressive disorder, and cognitive deficiency^66, 117, 118^
2	*MEMO1*	25	31896397	32139202	9.20e-04	—
12	*ALX1*	16	84148166	84269692	9.30e-04	Autism spectrum disorders^119, 120^
7	*GPER1*	36	1042968	1149977	9.40e-04	Anxiety and stress, bipolar disorder, and antipsychotic treatment for schizophrenia^121–124^

Start and end positions include default flanking regions of ±50 Kb from 5′ and 3′ UTRs of each gene, defined by default by VEGAS software (http://gump.qimr.edu.au/VEGAS/), and are based on UCSC annotation, build NCBI36/hg18 (Mar. 2006).

*An empirical P-value of 0 (from 10^6^ simulations) can be interpreted as P-value < 10^6^, which exceeds a Bonferroni-corrected threshold set at P-value < 2.8e-06 (~0.05/17,787).

We further tested the 40 genes surpassing the follow-up threshold of P-value < 1e-03 in an independent dataset from the first large-scale meta-analysis of ADHD GWAS, consisting of 2,064 ADHD trios, 896 ADHD cases and 2,455 controls from the Psychiatric Genetics Consortium^7, 22^. No signal was associated with ADHD after Bonferroni correction. Nominal signals, however, were found for *FBXO33* (P-value = 0.047 when considering all SNPs in the gene locus and P-value = 0.01 when considering the top 10% SNPs) and C15orf53 (P-value = 0.036 when considering the top 10% SNPs in the gene locus) (see Supplementary Table S1).

### Functional and Pathway Analysis

**Figure 1:**
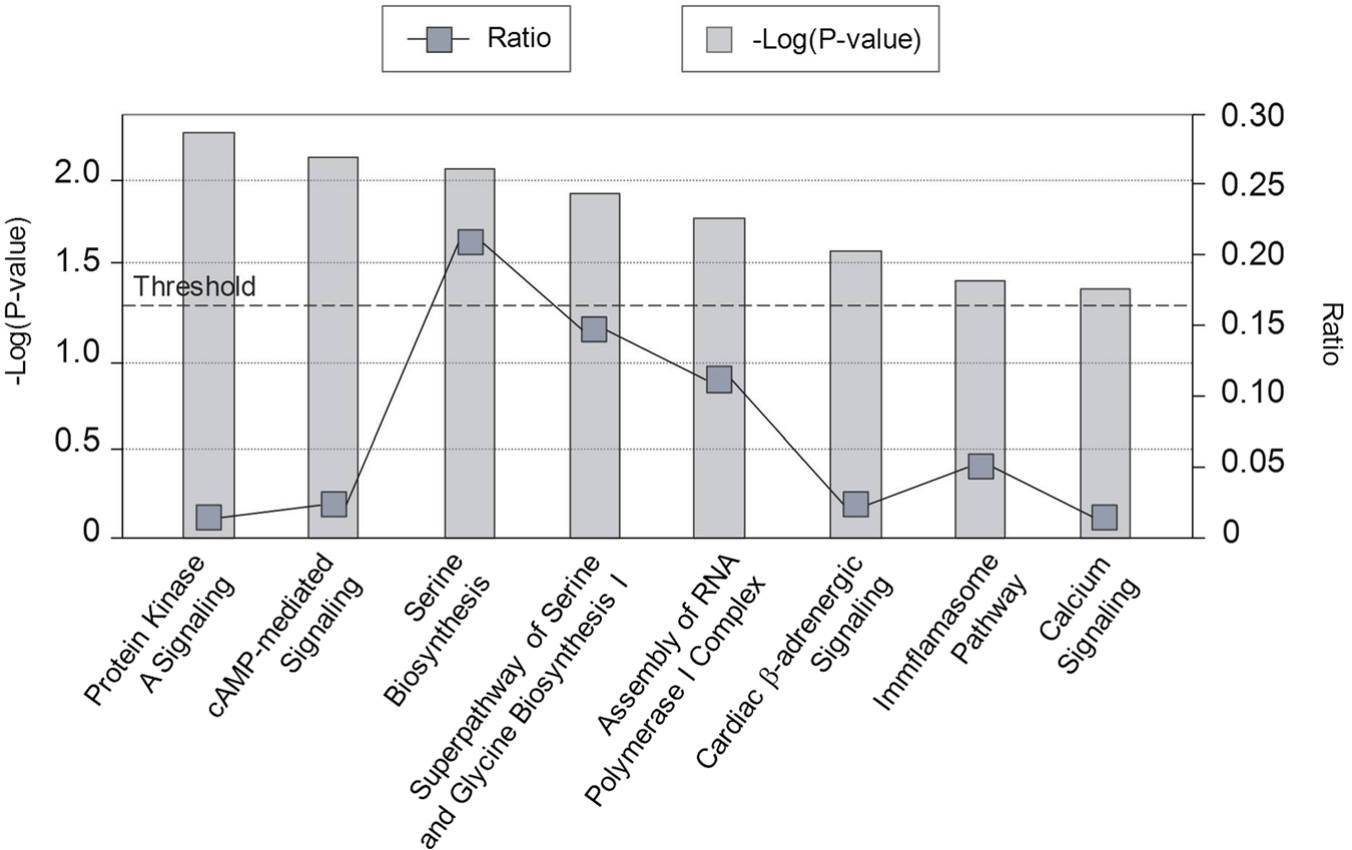
Enriched canonical pathways (P-value < 0.05) considering 40 genes from the gene-wide analyses surpassing follow-up significance threshold (P-value < 1e-03). The −log(P-value) from the Fisher’s exact test for each enriched category is indicated by grey bars. The ratio indicates the number of genes tested that map to the canonical pathway divided by the total number of genes that map to that pathway, and is represented by dark grey squares.

Although none of them survived BH multiple-testing correction, enrichment for eight canonical pathways was detected when the 40 genes from the two gene-sets identified in the gene-wide association analysis were considered jointly, including protein kinase A (PKA) and cAMP-mediated signaling (P-value = 5.75e-03 and P-value = 7.94e-03, respectively), serine biosynthesis and superpathway of serine and glycine biosynthesis (P-value = 9.33e-03 and P-value = 1.29e-02, respectively), assembly of RNA polymerase I complex (P-value = 1.66e-02), cardiac adrenergic signaling (P-value = 2.57e-02), inflammasome pathway (P-value = 3.80e-02) and calcium signaling (P-value = 4.27e-02) (Fig. 1). Among the top functions and diseases, most enriched categories were mainly related to organismal and embryonic development (P-value = 2.58e-04–4.00e-02), cell-to-cell signaling and interaction (P-value = 3.96e-04–3.47e-02) and cellular movement (P-value = 1.28e-03–3.60e-02) (Table 2). In addition, several nervous system-related terms and psychological traits were also identified among enriched categories, such as relaxation of mice (P-value = 5.55e-03), swelling of neurites (P-value = 7.40e-03), axonal transport of vesicles (P-value = 9.24e-03), abnormal morphology of nervous system (P-value = 1.10e-02) or addiction behaviour (P-value = 4.36e-02) (Table 3). Three relevant networks that included genes mainly related to cellular development, organization, function and maintenance, cell death and survival, and cell-to-cell signaling and interaction were generated (Table 4). The two most relevant networks were highly scored (P-score = 44 and P-score = 26, respectively) and included 18 (45%) and 12 (30%) out of the 40 genes considered for this analysis.

**Table 2:** Top-ten enriched super-categories from disease and functional enrichment analyses using IPA software, considering 40 genes identified in the gene-wide analyses surpassing follow-up significance threshold (P-value < 1e-03).

Super-category	P-value	Molecules
Embryonic Development	2.58e-04–3.83e-02	*NCSTN*, *ALX1*, *CSF2RB*, *PNN*, *MAB21L2*, *PEA15*
Organismal Development	2.58e-04–4e-02	*NCSTN*, *GPER1*, *ALX1*, *COPA*, *ATP1A1*, *CSF2RB*, *AKAP8*, *RAP1B*, *MAB21L2*, *NLRC4*, *PEA15*
Tissue Development	2.58e-04–4.94e-02	*NCSTN*, *GPER1*, *ALX1*, *CSF2RB*, *RAP1B*, *IPP*, *MAB21L2*
Cell-To-Cell Signaling and Interaction	3.96e-04–3.47e-02	*NCF4*, *CCL26*, *CSF2RB*, *STXBP3*, *RAP1B*
Cellular Movement	1.28e-03–3.65e-02	*NCF4*, *CCL26*, *GPER1*, *CSF2RB*, *RAP1B*, *NLRC4*
Hematological System Development and Function	1.28e-03–4.89e-02	*CCL26*, *NCF4*, *NCSTN*, *CSF2RB*, *RAP1B*, *NLRC4*
Immune Cell Trafficking	1.28e-03–3.65e-02	*NCF4*, *CCL26*, *CSF2RB*, *RAP1B*, *NLRC4*
DNA Replication, Recombination, and Repair	1.51e-03–2.39e-02	*GPER1*, *AKAP8*, *PEA15*
Cardiovascular System Development and Function	1.85e-03–2.39e-02	*AKAP10*, *GPER1*, *ATP1A1*, *CSF2RB*, *RAP1B*
Cell Cycle	1.85e-03–3.65e-02	*PEX19*, *PNN*, *NLRC4*, *PEA15*

**Table 3:** Significant nervous system-related categories identified in the disease and functional enrichment analyses using IPA software, considering 40 genes identified in the gene-wide analyses surpassing follow-up significance threshold (P-value < 1e-03).

Super-Categories	Diseases or Functions Categories	P-value	Molecules
Hereditary Disorder, Neurological Disease, Organismal Injury and Abnormalities	autosomal dominant spastic paraplegia type 4	1.85e-03	*SPAST*
Developmental Disorder, Hereditary Disorder, Neurological Disease, Organismal Injury and Abnormalities	autosomal recessive mental retardation type 39	1.85e-03	*TTI2*
Developmental Disorder, Neurological Disease, Organismal Injury and Abnormalities	meroanencephaly	1.85e-03	*ALX1*
Organismal Development	relaxation of mice	5.55e-03	*ATP1A1*
Connective Tissue Disorders, Developmental Disorder, Neurological Disease, Skeletal and Muscular Disorders	acrania	7.40e-03	*ALX1*
Cell Morphology, Cellular Assembly and Organization, Cellular Development, Cellular Function and Maintenance, Cellular Growth and Proliferation, Neurological Disease, Organismal Injury and Abnormalities	swelling of neurites	7.40e-03	*SPAST*
Nervous System Development and Function	antinociception of spinal cord	9.24e-03	*GPER1*
Cellular Assembly and Organization, Cellular Function and Maintenance, Nervous System Development and Function	axonal transport of vesicles	9.24e-03	*SPAST*
Nervous System Development and Function	abnormal morphology of nervous system	1.10e-02	*ALX1*, *MAB21L2*, *PEA15*, *RAP1B*, *SPAST*, *STXBP3*
Connective Tissue Disorders, Developmental Disorder, Organismal Development, Skeletal and Muscular Disorders	clefting of face	1.29e-02	*ALX1*
Embryonic Development, Nervous System Development and Function, Organismal Development, Tissue Morphology	abnormal morphology of neural crest	1.47e-02	*NCSTN*
Cell Morphology, Nervous System Development and Function, Tissue Morphology	abnormal morphology of neurites	3.92e-02	*RAP1B*, *SPAST*
Connective Tissue Development and Function, Organ Morphology, Organismal Development, Skeletal and Muscular System Development and Function, Tissue Development	abnormal morphology of supraoccipital bone	4.00e-02	*ALX1*
Behavior	addiction behaviour	4.36e-02	*ATP1A1*
Cell Morphology, Nervous System Development and Function, Neurological Disease, Tissue Morphology	loss of axons	4.89e-02	*RAP1B*

**Table 4:** Significant generated networks (P-score < 8) using IPA software, considering 40 genes identified in the gene-wide analyses surpassing follow-up significance threshold (P-value < 1e-03).

Molecules in Network	P-score*	Focus Molecules	Top Diseases and Functions
*ABL1*, ***AKAP8***, ***AKAP10***, *Akt*, ***ATP1A1***, *C1QBP*, *caspase*, ***CASQ1***, ***CCL26***, ***COPA***, ***CSF2RB***, *DDX3X*, *EMILIN1*, *ERK*, *ERK1/2*, *Gpcr*, ***GPER1***, ***GPR146***, *Insulin*, ***MAST2***, ***MEMO1***, ***MLNR***, ***NCF4***, ***NCSTN***, *NFkB (complex)*, ***NLRC4***, *P38 MAPK*, ***PEA15***, *PI3K (complex)*, *Pka*, *Pkc(s)*, ***RAP1B***, ***STXBP3***, *TARDBP*, *TRAF6*	44	18	Cell-To-Cell Signaling and Interaction, Cellular Development, Hematological System Development and Function
*AES*, *APP*, *C3orf33*, *CEP57*, *Collagen type VII*, ***CTAGE5***, ***DCAF8***, *DGCR6/LOC102724770*, ***DUSP26***, *ELAVL1*, *EMILIN1*, *FAM20B*, ***FBXO33***, ***GPBP1L1***, *HIGD2A*, *HINT3*, *HSP90AB1*, ***MAB21L2***, *MAPK3*, *MMP3*, ***PEX19***, *PEX26*, *PLD6*, ***PNN***, *RAF1*, *RBX1*, ***RIN2***, ***RNF122***, *SELT*, ***SPAST***, *TARDBP*, *TMTC4*, *TRAF6*, ***TTI2***, *YBX1*	26	12	Cellular Assembly and Organization, Cellular Compromise, Cellular Function and Maintenance
*ABL1*, *Actin*, *ALB*, ***ALX1***, *BZW2*, ***CALML4***, *Cdc2*, *CDC25A*, *CREB1*, *CUL1*, *CYBA*, *Cyclin B*, *EGLN1*, *EPO*, *GABBR1*, *GDF15*, *HNF1A*, *IKBKB*, ***IPP***, *IRF3*, *KHDRBS1*, *KRT8*, *KRT18*, *LANCL1*, ***MIA2***, *mir-296*, *MYB*, *MYBL2*, *MYO5B*, ***NAA20***, ***TAF1C***, *TARDBP*, *TGFB1*, *TRAF6*, *YBX1*	11	6	Cell Death and Survival, Digestive System Development and Function, Hepatic System Development and Function

Focus molecules from the follow-up gene-set are shown in bold.

^*^P-score = −log10(P-value).

### Gene expression analysis in peripheral blood mononuclear cells (PBMCs)

Expression levels of the 40 genes in the two gene-sets identified in the gene-wide association study were explored in microarray data from peripheral blood mononuclear cells (PBMCs) of 45 adults with ADHD and 39 healthy controls. No data were available for two of them (*WDR42A* and *NAT5*) and another five were excluded because the corresponding probes matched to multiple genes (*COPA*, *TMEM69*, *TAF1C*, *MEMO1*, *C8orf41*). Although no significant expression differences were identified after Bonferroni correction, tentative evidence for overexpression was detected in ADHD subjects for *STXBP3* (P-value = 1.9e-03; log fold change = 0.115) and *RNF122* (P-value = 0.045; log fold change = 0.111) and decreased expression for *MAB21L2* (P-value = 0.036; log fold change = −0.77) when compared to controls (Table 5). Differences in *RNF122*, *STXBP3* and *MAB21L2* expression levels were subsequently tested by RT-qPCR and evidence for significant overexpression was confirmed for *RNF122* (but not for *STXBP3* or *MAB21L2*) after controlling for multiple testing using the Bonferroni correction (P-value = 3.04e-03; OR = 4.13 [2.46–5.81]); Table 6).

**Table 5:** Differentially expressed genes in peripheral blood mononucleated cells (PBMCs) of 45 pharmacologically-naive ADHD subjects and 39 healthy unrelated controls in the microarray analysis.

Probe ID	Log-Fold change	P-value	Gene Symbol	Description
8150186	0.111	0.048	*RNF122*	Localizes to the endoplasmic reticulum and golgi apparatus and may be associated with cell viability by inducing necrosis and apoptosis. Mediates protein-protein and protein-DNA interactions, and has been identified as a new E3 ubiquitin ligase that can ubiquitinate itself and undergo degradation in a RING finger-and proteasome-dependent manner^41^.
7903541	0.115	2.3e-03	*STXBP3*	Plays an integral role in vesicle transport through their interaction with SNAREs and could play a positive regulatory role in SNARE assembly^125^.
8097773	-0.77	0.034	*MAB21L2*	Required for several aspects of embryonic development including normal development of the eye. May be involved in neural development^80^.

**Table 6:** Validation of *MAB21L2*, *STXBP3* and *RNF122* gene expression differences between pharmacologically-naive ADHD subjects and healthy unrelated controls by RT-qPCR.

Gene name	Gene Symbol	Gene ID	Location	Assay ID	Amplicon Size		
**Assay information of target and control genes**							
**Housekeeping gene**							
growth arrest and DNA damage inducible alpha	*GADD45A*	1647	1p31.2	Hs00169255_m1	123		
**Target genes**							
mab-21-like 2	*MAB21L2*	10586	4q31	Hs00740710_s1	93		
syntaxin binding protein 3	*STXBP3*	6814	1p13.3	HS01029364_m1	149		
ring finger protein 122	*RNF122*	79845	8p12	Hs00227141	99		
**Target gene**	**Control gene**	**ODDS Ratio**	**Standard Error**	**Z-value**	**P-value**	**Number of controls**	**Number of cases**
**Results from RT-qPCR validation of** ***MAB21L2***, ***STXBP3*** **and** ***RNF122*** **genes**							
*RNF122*	*GADD45A*	4.133	0.517	2.744	**3**.**04e-03**	34	42
*STXBP3*	*GADD45A*	2.361	0.521	1.648	0.049	40	44
*MAB21L2*	*GADD45A*	0.866	0.701	−0.206	0.419	35	25

Statistically significant P-values after applying a Bonferroni threshold of P-value < 0.017, when taking into account three genes (0.05/3), are shown in bold.

### *Cis*-expression Quantitative Trait Loci (*cis*-eQTL) Analyses and Prediction of Functional Effects

To better define the role of *RNF122* in ADHD and to detect potential functional variants, we imputed markers at this locus in the original dataset of 603 subjects with ADHD and 583 healthy controls and found 47 out of 138 SNPs nominally associated with ADHD. Although none of them exceeded the conservative Bonferroni-corrected threshold, three surpassed the SNPSpD multiple-testing correction (rs3735951, rs9297208 and rs9297209) (See Supplementary Figure S1). Suggestive evidence for association was detected along the entire gene, with the top signal at the genotyped variant rs3735951, located in exon 2, being the allele T the risk one (P-value = 8.18e-04; OR = 1.37[1.14–1.65]) (See Supplementary Figure S1). After conditional analysis, no evidence for additional independent effects was detected along the gene, being the entire association of this locus explained by the rs3735951 marker (See Supplementary Table S2). Although we did not find evidence for rs3735951 acting as *cis*-eQTL in preexisting datasets of cortical gene expression (GSE8919 and GSE30272)^35, 36^, that could explain the *RNF122* expression differences identified in ADHD (See Supplementary Table S3), functional prediction revealed that rs3735951 may lie within a partially overlapped exonic splicing enhancer (ESE) site and an exonic splicing silencer (ESS) site (See Supplementary Table S4). While the rs3735951T risk allele may create an ESE site potentially targeted by the spliceosome factors SRSF2 and SRSF5, the rs3735951C allele may generate an ESS site (See Supplementary Table S4).

## Discussion

With the aim of uncovering new underlying genes involved in persistent ADHD and providing additional evidence for the contribution of previously identified genes, we performed gene-wide and pathway enrichment analyses in a pre-existing GWAS dataset of adult ADHD followed by gene expression profiling. Our results provide preliminary evidence for genetic association between ADHD and the *RNF122* gene and abnormal *RNF122* expression levels in PBMCs of medication-naive ADHD subjects. These findings highlight *RNF122* as a strong candidate for ADHD.

*RNF122* (RING Finger Protein 122) is an E3 ubiquitin ligase involved in the proteasome-mediated processing, trafficking, and degradation of proteins that acts as an essential mediator of the substrate specificity of ubiquitin ligation^41^. This finding is in line with previous genome-wide analyses supporting the involvement of genes related to the ubiquitination machinery in the genetic susceptibility to ADHD or attention function, including the *FBXO33* and *PARK2* genes, which also encode components of the E3 ubiquitin-protein ligase complex^12, 42^. Interestingly, the second most significant network identified in the present study revealed an indirect connection between *RNF122* and *FBXO33*, and included other best hits such as *MAB21L2* or *PEX19* (Tables 1 and 3). The ubiquitin-proteasome pathway controls wide-ranging functions in the central nervous system (CNS), including fine-tuning of synaptic connections during development and synaptic plasticity in the adult organism, and has been identified as a well-founded pathway for other psychiatric or neurological conditions, including bipolar disorder, schizophrenia, Alzheimer’s disease, Parkinson’s disease, Huntington’s disease, intellectual disability or autism spectrum disorder^43–50^. In line with these findings, the identification of “Protein Kinase A Signaling” and “cAMP-mediated Signaling” as the two most significantly enriched canonical pathways may also support the role of the identified genes in adult synaptic plasticity mediated by the ubiquitin-proteasome system since ubiquitination, degradation and subsequent removal of regulatory subunits of protein Kinase A in response to cAMP stimulation induce neuronal differentiation and activity, as well as synaptic plasticity (Fig. 1)^43, 44, 52, 53^.

The overexpression of *RNF122* that we found in persistent ADHD is in agreement with previous studies reporting increased expression levels of E3 ubiquitin ligases in neuropsychiatric disorders^51, 54^. Given its role in synaptic surface-protein turnover and in the regulation of the number of scaffolding proteins and neurotransmitter receptors, increased ubiquitin ligation and subsequent enhanced protein removal may compromise neuronal functioning^43, 55, 56^. Although its consequences in the remodelling of the synaptic density should be further investigated, enhanced ubiquitin-proteasome pathway activity may entail downregulation of synaptic receptors, such as gamma-aminobutyric acid (GABA), α-amino-3-hydroxy-5-methyl-4-isoxazole propionic acid (AMPA), N-methyl-d-aspartate (NMDA) or nicotinic acetylcholine (nACh) receptors; and eventually lead to the neuropathological mechanisms that likely underlie neuropsychiatric disorders^51, 54, 56–60^.

We also identified a significant association between *CTAGE5* and persistent ADHD after Bonferroni correction. *CTAGE5*, together with other top hits identified in the gene-wide analyses, namely *FBXO33*, *MIA2* and *PNN*, lies within a genomic segment on chromosome 14 that was found to be deleted in three subjects with different neurologic and/or psychiatric traits, such as mild mental retardation, severe learning difficulties, motor alterations or ADHD symptoms^61^. *FBXO33* gene encodes a member of the F-box protein family which acts as a component of an E3 ubiquitin-protein ligase complex and is involved in targeting substrates for proteasomal degradation, while *CTAGE5* and *MIA2* genes are both involved in the traffic of large cargos from the endoplasmic reticulum to the Golgi apparatus, a process that involves ubiquitination^62^. Thus, together with the involvement of *RNF122* in ADHD, these findings highlight the need for further studies exploring in depth the potential role of the ubiquitin - proteasome pathway in the susceptibility to ADHD.

Additionally, nominal signals were identified for *C15orf53* and *FBXO33* in both the discovery and the replication datasets. Interestingly, *C15orf53* has previously been associated with bipolar disorder and alcohol dependence, and *FBXO33*, which may be involved in autism spectrum disorders, was highlighted in the previous SNP-based GWAS that we performed in the same discovery dataset and provides additional evidence supporting the ubiquitination machinery as a new mechanism for ADHD^7, 22^.

Our study should be viewed in light of several methodological considerations:

First, we attempted to overcome SNP-based GWAS limitations by using a gene-wide approach, a promising complement to GWAS since it considers the combined effects of all genetic variants within a locus and might be more powerful than traditional SNP-based strategies^9^. Our modest sample size and the anticipated small effect of common polymorphisms in complex traits, however, may have prevented us from detecting additional signals, apart from *CTAGE5*, exceeding the conservative Bonferroni correction. Additionally, although controls were screened retrospectively for ADHD symptoms, no specific scale or structured interview was used to discard the presence of other psychiatric disorders and, therefore, certain degree of heterogeneity in the control sample may exist and might influence our results.

Second, the conditional analysis highlighted rs3735951 as the top-ranked variant at the *RNF122* locus and eliminated evidence for association for other SNPs within the region. This sequence variant did not tag any eQTL for *RNF122* in human brain samples from two available pre-existing data sets^35, 36^, but lies within potential exonic splicing regulatory elements. Specifically, the rs3735951T risk allele is predicted to create an exonic splicing enhancer (ESE), potentially targeted by *SRSF2* and *SRSF5* spliceosome factors, which could result in alternative splicing of the *RNF122* transcript and, thus, modulate substrate specificity or ligation function (Supplementary Table S4)^37–40^. Given that rs3735951 lies within a LD block that spans the entire gene, however, we cannot discard additional relevant variants within the locus exerting functional effects.

Third, while we identified genes displaying biologically interesting functions and pathways pointing to the ubiquitin-proteasome pathway as a promising candidate system, no overlap was observed between our gene-wide results and previous findings in ADHD through either individual GWAS or meta-analysis^2–4, 8^. The replication attempt in an independent sample, however, yielded preliminary evidence for nominal association of ADHD with *FBXO33* and *C15orf53*. Heterogeneity between populations and differences in study design or in the proportion of persistent ADHD between datasets may account for discordant results across studies, making it difficult to establish direct comparisons between reports. For this reason, further replication of the gene-wide and gene expression results in independent cohorts are needed to confirm these associations and to estimate the magnitude of their effects.

Fourth, aberrant *RNF122* expression levels were detected in PBMCs of ADHD subjects, naive for pharmacological treatment. Transcriptome analysis in peripheral blood has become an increasingly useful tool in the search for biomarkers in multiple medical fields, including psychiatric disorders, given their great deal of potential for non-invasive screening, diagnosis and prognosis, or for differentiation of biological endophenotypes, development of targeted therapies and anticipation of clinical response or adverse effects^63^. Although whole blood shares substantial transcriptome similarities with different CNS tissues so as to use peripheral expression profiles as a surrogate for gene expression in the CNS, further evidence in brain tissues is required to assert the role of *RNF122* in the pathophysiology of ADHD^64^.

In conclusion, we performed gene-wide and pathway enrichment analyses using data from a pre-existing GWAS dataset of persistent ADHD and provided tentative evidence for the involvement of the *CTAGE5* and *RNF122* genes in the susceptibility to the disorder. We also detected overexpression of the *RNF122* gene in PBMCs of adult ADHD patients, placing this gene as a promising candidate for the disorder. The evidence provided by our findings point to the ubiquitin-proteasome system as a well-founded pathway involved in the etiology of ADHD. Further collaborative efforts are required to disentangle the exact molecular mechanisms by which *CTAGE5*, *RNF122*, and the ubiquitin-proteasome system may contribute to the pathophysiology of ADHD and other neuropsychiatric disorders.

## Electronic supplementary material

Supplementary information
