# Supplementary material for: Gene-wide Association Study Reveals RNF122 Ubiquitin Ligase as a Novel Susceptibility Gene for Attention Deficit Hyperactivity Disorder
Source: Sci Rep. 2017 Jul 14;7:5407. doi: 10.1038/s41598-017-05514-7 (PMC5511183; doi:10.1038/s41598-017-05514-7)

SUPPLEMENTARY INFORMATION

Gene-wide Association Study Reveals *RNF122* Ubiquitin Ligase as a Novel Susceptibility Gene for Attention Deficit Hyperactivity Disorder

[a]Iris Garcia-Martínez[1-2], [a]Cristina Sánchez-Mora[1-3], María Soler Artigas[1-3], Paula Rovira[1-2], Mireia Pagerols[1-2], Montse Corrales [2,4], Eva Calvo-Sánchez[1-2], Vanesa Richarte[2-4], Mariona Bustamante[5-8], Jordi Sunyer[5,7-9], Bru Cormand[10-13], Miquel Casas[1-4], Josep Antoni Ramos-Quiroga[1-4], ***Marta Ribasés**[1-3].

[1]Psychiatric Genetics Unit, Group of Psychiatry, Mental Health and Addiction, Vall d'Hebron Research Institute (VHIR), Universitat Autònoma de Barcelona, Barcelona, Catalonia, Spain.

[2]Department of Psychiatry, Mental Health and Addictions, Hospital Universitari Vall d'Hebron, Barcelona, Catalonia, Spain.

[3]Biomedical Network Research Centre on Mental Health (CIBERSAM), Instituto de Salud Carlos III, Madrid, Spain.

[4]Department of Psychiatry and Legal Medicine, Universitat Autònoma de Barcelona, Barcelona, Catalonia, Spain.

[5]ISGlobal, Centre for Research in Environmental Epidemiology (CREAL), Barcelona, Spain

[6]Genomics and Disease Group, Bioinformatics and Genomics Program, Centre for Genomic Regulation (CRG), Barcelona, Spain

[7]Universitat Pompeu Fabra (UPF), Barcelona, Spain

[8]CIBER Epidemiología y Salud Pública (CIBERESP), Madrid, Spain

[9]IMIM (Hospital del Mar Medical Research Institute), Barcelona, Spain

[10]Departament de Genètica, Microbiologia i Estadística, Facultat de Biologia, Universitat de Barcelona, Barcelona, Catalonia, Spain.

[11]Centro de Investigación Biomédica en Red de Enfermedades Raras (CIBERER), Instituto de Salud Carlos III, Madrid, Spain.

[12]Institut de Biomedicina de la Universitat de Barcelona (IBUB), Barcelona, Catalonia, Spain.

[13]Institut de Recerca Sant Joan de Déu (IR-SJD), Esplugues, Catalonia, Spain

[a]Equally contributed.

*Correspondence: **Marta Ribasés**, Psychiatric Genetics Unit, Vall d'Hebron Research Institute, Passeig Vall d'Hebron 119-129, 08035 Barcelona, Spain; e-mail: marta.ribases@vhir.org

CONTACT INFORMATION

Iris Garcia-Martínez
e-mail: iris.garcia@vhir.org
Psychiatric Genetics Unit
Vall d'Hebron Institut de Recerca
Passeig Vall d'Hebron, 119-129
08035 Barcelona
SPAIN
Phone: +34 93 274 6734
Fax: +34 93 489 4587

Cristina Sánchez-Mora
e-mail: cristina.sanchez@vhir.org
Psychiatric Genetics Unit
Vall d'Hebron Institut de Recerca
Passeig Vall d'Hebron, 119-129
08035 Barcelona
SPAIN
Phone: +34 93 274 6734
Fax: +34 93 489 4587

Maria Soler
e-mail: maria.soler@vhir.org
Psychiatric Genetics Unit
Vall d'Hebron Institut de Recerca
Passeig Vall d'Hebron, 119-129
08035 Barcelona
SPAIN
Phone: +34 93 274 6734
Fax: +34 93 489 4587

Paula Rovira
e-mail: paula.rovira@vhir.org
Psychiatric Genetics Unit
Vall d'Hebron Institut de Recerca
Passeig Vall d'Hebron, 119-129
08035 Barcelona
SPAIN
Phone: +34 93 274 6734
Fax: +34 93 489 4587

Mireia Pagerols
e-mail: mireia.pagerols@vhir.org
Psychiatric Genetics Unit
Vall d'Hebron Institut de Recerca
Passeig Vall d'Hebron, 119-129
08035 Barcelona
SPAIN
Phone: +34 93 274 6734
Fax: +34 93 489 4587

Montse Corrales
e-mail: mcorrales@vhebron.net
Department of Psychiatry
Hospital Universitari Vall d'Hebron
Passeig Vall d'Hebron, 119-129
08035 Barcelona
SPAIN
Phone: +34 93 489 4295
Fax: +34 93 489 4587

Eva Calvo
e-mail: eva.calvo@vhir.org
Psychiatric Genetics Unit
Vall d'Hebron Institut de Recerca
Passeig Vall d'Hebron, 119-129
08035 Barcelona
SPAIN
Phone: +34 93 274 6734
Fax: +34 93 489 4587

Vanesa Richarte
e-mail: vricharte@vhebron.net
Department of Psychiatry
Hospital Universitari Vall d'Hebron
Passeig Vall d'Hebron, 119-129
08035 Barcelona
SPAIN
Phone: +34 93 489 4295
Fax: +34 93 489 4587

Mariona Bustamante
e-mail: mariona.bustamante@isglobal.org
ISGlobal, Centre for Research in Environmental Epidemiology (CREAL)
Parc de Recerca Biomedica de Barcelona, PRBB
Carrer Dr. Aiguader, 88
08003 Barcelona
SPAIN
Phone: +34 93 316 01 97

Jordi Sunyer
e-mail: jordi.sunyer@isglobal.org
ISGlobal, Centre for Research in Environmental Epidemiology (CREAL)
Parc de Recerca Biomedica de Barcelona, PRBB
Carrer Dr. Aiguader, 88
08003 Barcelona
SPAIN
Phone: +34 93 214 73 50

Bru Cormand
e-mail: bcormand@ub.edu
Departament de Genètica, Microbiologia i Estadística
Facultat de Biologia, Universitat de Barcelona
Avinguda Diagonal, 643
08028 Barcelona
SPAIN
Phone: +34 93 402 1013
Fax: +34 93 403 4420

Miquel Casas
e-mail: mcasas@vhebron.net
Department of Psychiatry
Hospital Universitari Vall d'Hebron
Passeig Vall d'Hebron, 119-129
08035 Barcelona
SPAIN
Phone: +34 93 489 4295
Fax: +34 93 489 4587

Josep Antoni Ramos-Quiroga
e-mail: jaramos@vhebron.net
Department of Psychiatry
Hospital Universitari Vall d'Hebron
Passeig Vall d'Hebron, 119-129
08035 Barcelona
SPAIN
Phone: +34 93 489 4294
Fax: +34 93 489 4587

Marta Ribasés
e-mail: marta.ribases@vhir.org
Psychiatric Genetics Unit
Vall d'Hebron Institut de Recerca
Passeig Vall d'Hebron, 119-129
08035 Barcelona
SPAIN
Phone: +34 93 274 6734
Fax: +34 93 489 4587

Supplementary Table S1. Gene-wide replication test by VEGAS2 software in an independent sample of 2,064 ADHD trios, 896 ADHD cases and 2,455 controls considering the 40 genes identified in discovery sample.

Chr	Gene	Approach	Start	Stop	P-value	Best SNP	SNP pvalue
14	*FBXO33*	All SNPS	38886709	39021371	**0.0479**	rs1955716	3.1e-04
14	*FBXO33*	Top 10% SNPs	38886709	39021371	**0.0102**	rs1955716	3.1e-04
15	*C15orf53*	All SNPS	36726090	36829531	0.0899	rs7495764	3.2e-04
15	*C15orf53*	Top 10% SNPs	36726090	36829531	**0.0360**	rs7495764	3.2e-04

Bold denotes nominal significance at P-value<0.05.

Start and stop positions include flanking regions of ± 50 Kb from 5' and 3' UTRs of each gene, and are based on UCSC annotation, build NCBI36/hg18 (Mar. 2006).

Supplementary Table S2. Top-20 SNPs from the association analysis within *RNF122* locus conditioned by rs3735951 in 603 subjects with persistent ADHD and 583 healthy unrelated controls.

CHR	SNP	BP	A1	NMISS	OR	STAT	P-value
8	rs72629435	33433372	A	991	4.047	1.774	7.61e-02
8	rs7841080	33428277	G	996	1.377	1.746	8.09e-02
8	rs183029091	33432508	G	1009	6.445	1.734	8.29e-02
8	rs80189016	33425679	C	999	2.1	1.702	8.88e-02
8	rs78440613	33413413	T	1006	1.827	1.699	8.94e-02
8	chr8_33409927_D	33409927	D	1006	1.742	1.619	1.05e-01
8	rs12678327	33401165	G	1005	1.702	1.548	1.22e-01
8	rs61533906	33401433	T	1005	1.702	1.548	1.22e-01
8	rs80051675	33401979	T	1005	1.702	1.548	1.22e-01
8	rs75858714	33403811	T	1006	1.698	1.541	1.23e-01
8	rs76470057	33407342	C	1007	1.694	1.533	1.25e-01
8	rs74782031	33412433	C	1007	1.694	1.533	1.25e-01
8	rs61212882	33412642	A	1006	1.686	1.52	1.29e-01
8	rs58644314	33412681	A	1006	1.686	1.52	1.29e-01
8	rs116970247	33416003	C	1001	0.6962	-1.52	1.29e-01
8	rs146756167	33434272	A	997	3.223	1.501	1.33e-01
8	rs78795401	33418343	A	1011	1.673	1.497	1.35e-01
8	rs79853173	33419218	A	1011	1.673	1.497	1.35e-01
8	rs75427835	33419701	G	1011	1.673	1.497	1.35e-01
8	rs145082055	33432965	A	944	1.901	1.483	1.38e-01

Abbreviations: **CHR:** chromosome, **SNP:** single nucleotide polymorphism, **BP:** base pair (chromosomal position in Hg19), **A1:** tested allele from PLINK (by default, minor allele), **NMISS:** number of non-missing individuals included in analysis; **OR:** odds ratio, **STAT:** coefficient t-statistic, **P-value:** asymptotic P-value for t-statistic.

Supplementary Table S3. Results from *cis*-eQTL analyses considering rs3735951 and human cortical gene expression data from Myers *et al.* and Colantuoni *et al.* [35-36]. Covariates were included in the model when their association with the outcome surpassed a threshold of P-value<0.2. The P-values for the covariates do not represent the test for the SNP-phenotype association after controlling for the covariate. The covariate term is the test associated with the covariate-phenotype association.

**Myers *et al*. human cortical gene expression data GSE8919 [33]**								
**CHR**	**SNP**	**BP**	**A1**	**TEST**	**NMISS**	**BETA**	**STAT**	**P-value**
8	rs3735951	33416222	C	ADD	57	-0.081	-0.601	0.549
8	rs3735951	33416222	C	gender	57	0.597	3.078	3.32e-03
8	rs3735951	33416222	C	age_at_death	57	-0.032	-2.587	1.25 e-02
8	rs3735951	33416222	C	transcripts_detected_rate24354	57	-16.83	-6.769	1.16e-08
**Colantuoni *et al*. human cortical gene expression data GSE30272 [34]**								
**CHR**	**SNP**	**BP**	**A1**	**TEST**	**NMISS**	**BETA**	**STAT**	**P-value**
8	rs3735951	33416222	C	ADD	92	-0.016	-0.100	0.920
8	rs3735951	33416222	C	sv2	92	2.804	1.766	8.09e-02

Abbreviations: **CHR:** chromosome, **SNP:** single nucleotide polymorphism identifier, **BP:** base pair (chromosomal position in Hg19), **A1:** tested allele from PLINK (by default, minor allele), **TEST:** code for the test (ADD=additive linear association; else=covariates), **NMISS:** number of non-missing individuals included in analysis; **BETA:** regression coefficient calculated with A2 as reference allele, **STAT:** coefficient t-statistic, **P-value:** asymptotic p-value for t-statistic.

Supplementary Table S4. Prediction of functional effects for rs3735951 using SNPinfo and ESEfinder servers.

**SNPinfo**				
**Exonic Splicing Enhancer (ESE) or Exonic Splicing Silencer (ESS)**				
**SNP**	**Allele**	**Forward Sequence**	**Prediction Method**	**Binding Site**
rs3735951	C	TC**C**TGG	FAS-ESS	ESS
rs3735951	T	**T**TGGAA	RESCUE-ESE	ESE

**ESEfinder**			
**Splicing Factor**	**ESE motif**	**Score**	**Threshold**
SRSF2	**C**TGGAAAC	3.98110	2.383
SRSF5	C**C**TGGAA	3.61680	2.67
SRSF2	**T**TGGAAAC	3.53120	2.383
SRSF5	C**T**TGGAA	3.04897	2.67

Supplementary Figure S1. Regional association plot including the *RNF122* locus on chromosome 8p12 and considering imputed genotype data using the 1000 Genomes Project dataset with the Ricopili pipeline. The x-axis shows physical distance (kb) and y-axis shows -Log10(P-value) values in 603 subjects with persistent ADHD and 583 healthy unrelated controls. The colour reflects nominal significance threshold for each tested SNP, being black dots associated markers (P-values< 0.05), and grey dots, not associated SNPs (P-values>0.05). The most associated SNP, rs3735951, is shown in diamond.

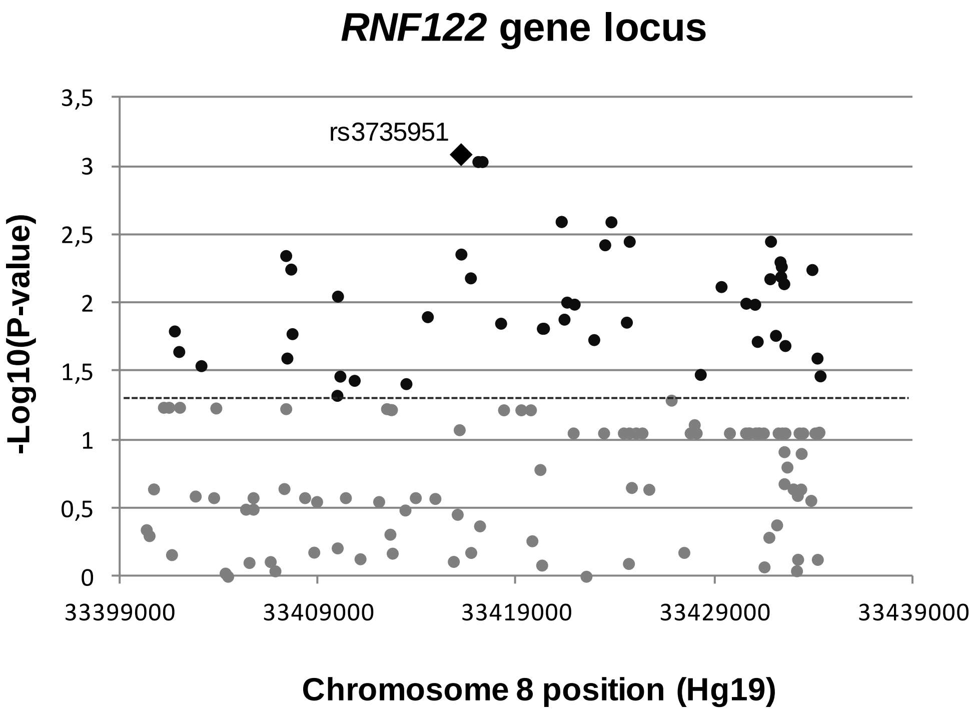

Supplement: Supplementary file 1 — Supplementary information [file 41598_2017_5514_MOESM1_ESM.pdf]
